# Supplementary material for: A summer in the greater Paris: trophic status of peri-urban lakes shapes prokaryotic community structure and functional potential
Source: Environ Microbiome. 2025 Feb 17;20:24. doi: 10.1186/s40793-025-00681-x (PMC11834611; doi:10.1186/s40793-025-00681-x)
Supplement: Supplementary file 1 — Supplementary Material 1 [file 40793_2025_681_MOESM1_ESM.docx]

**Supplementary figures**

**
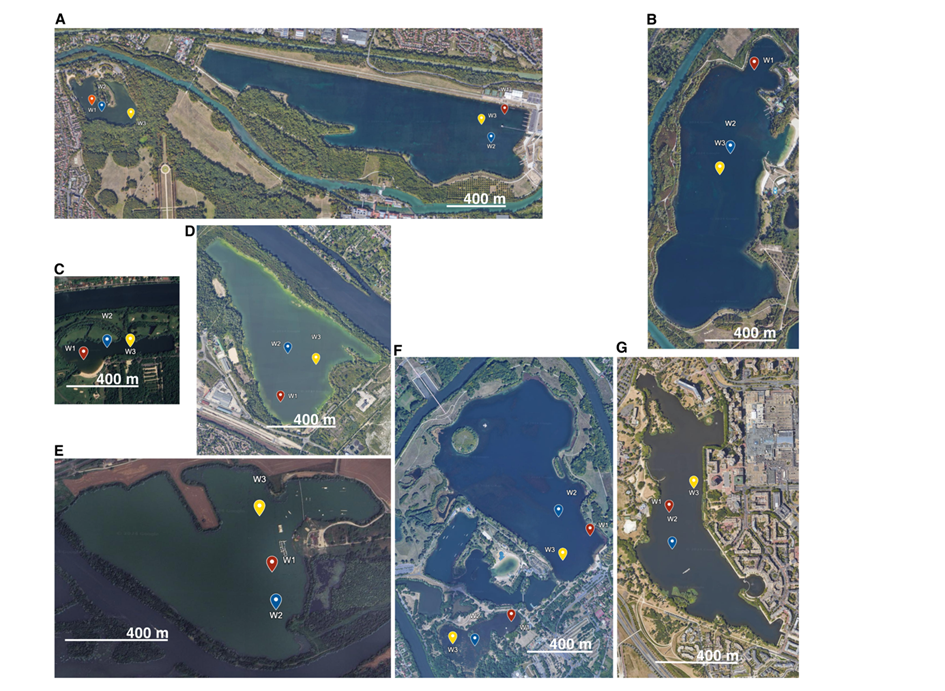
**

**Fig. S1: Satellite view of the lakes.** Images were taken from Google Earth © displaying the three sampling coordinates within each lake (see **Table S1.1** and **S1.2** for coordinates) : CSM (**A**, on the left; 3.6 m depth and 9.7 ha), VSM (**A**, on the right; 4.7 m depth and 86.7 ha), JAB (**B**; 7.4 m depth and 76.9 ha), BLR (**C**; 2.6 m depth and 7.3 ha), VSS (**D**; 4.9 m depth and 45.8 ha), GDP (**E**; 3.75 m depth and 51.9 ha), CER-S (lower **F**; 2.5 m depth and 10.5 ha), CER-L (upper **F**; 5.75 m depth and 91.0 ha) and CRE (**G**; 5.1 m depth and 40.1 ha).

**
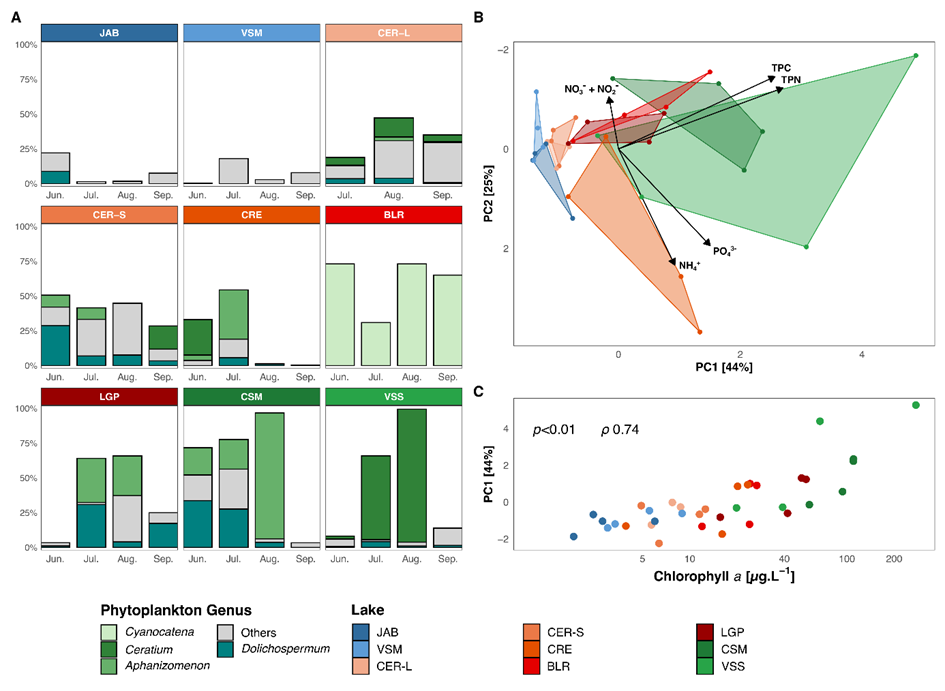
**

**Fig. S2: Supplementary analysis of abiotic and biotic parameters.
A**: Median relative biovolume of *Ceratium* (Miozoa) and all Cyanobacterial genera (grouped in “Others” if not affiliated to *Cyanocatena*, *Dolichospermum* or *Aphanizomenon*, 104 samples). **B:** PCA plot based on nutrients parameters (TPC, TPN, PO_4_^3-^, NH_4_^+^, NO_3_^-^+NO_2_^-^, Table S2). Polygons represent the maximal area delimited by samples coordinates for each lake. **C:** Relationship between the Chl*a* concentration and the PC1 coordinates, assessed with a Spearman correlation. Lakes are colored according to their trophic status (see Fig. 1B).

**
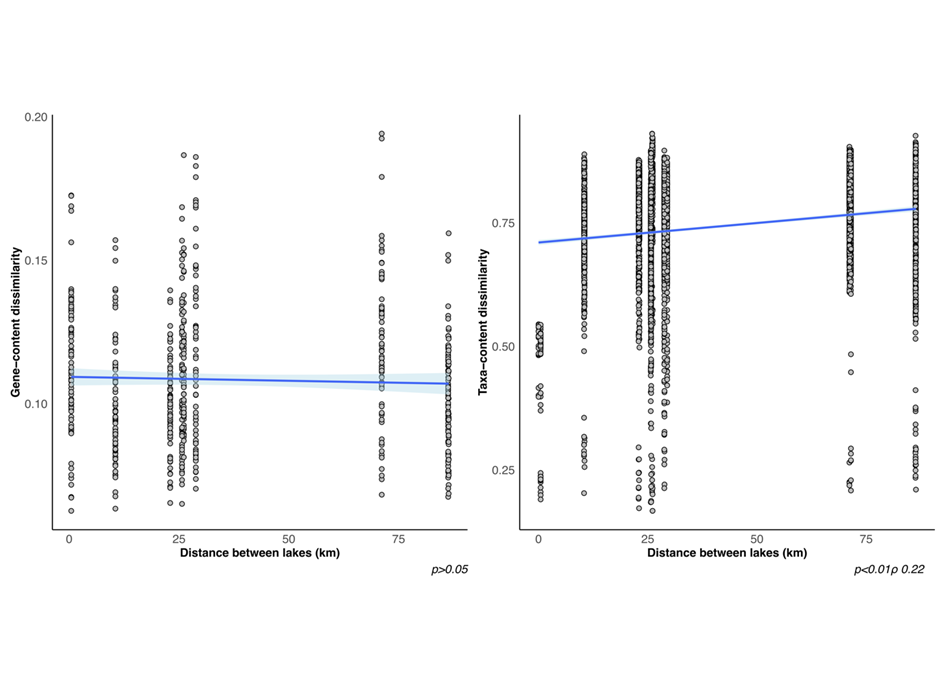
**

**Fig. S3: Influence of the spatial distance between lakes on the gene- and taxa- content dissimilarities. A and B:** Relationship between the gene- (KOs, 35 samples, **A**) and taxa-content (ASVs, 104 samples, **B**) dissimilarities (BC) and the distances between lakes (in km). Relationship significances are assessed by Spearman correlation.

**
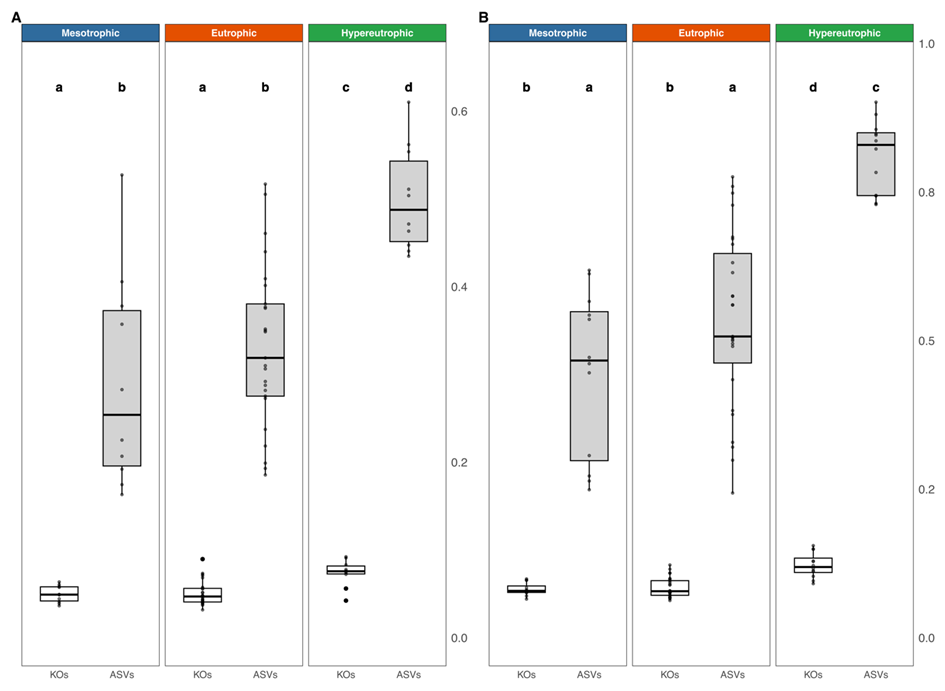
**

**Fig. S4:** Intra-seasonal heterogeneity (**A**) and overall dissimilarity value ranges (**B**) based on gene-content (KOs, white boxplots) and taxa-content (ASVs, grey boxplots) BC dissimilarity for each lake according to their trophic status. Letters indicate the significance of each trophic status (LMM).

**
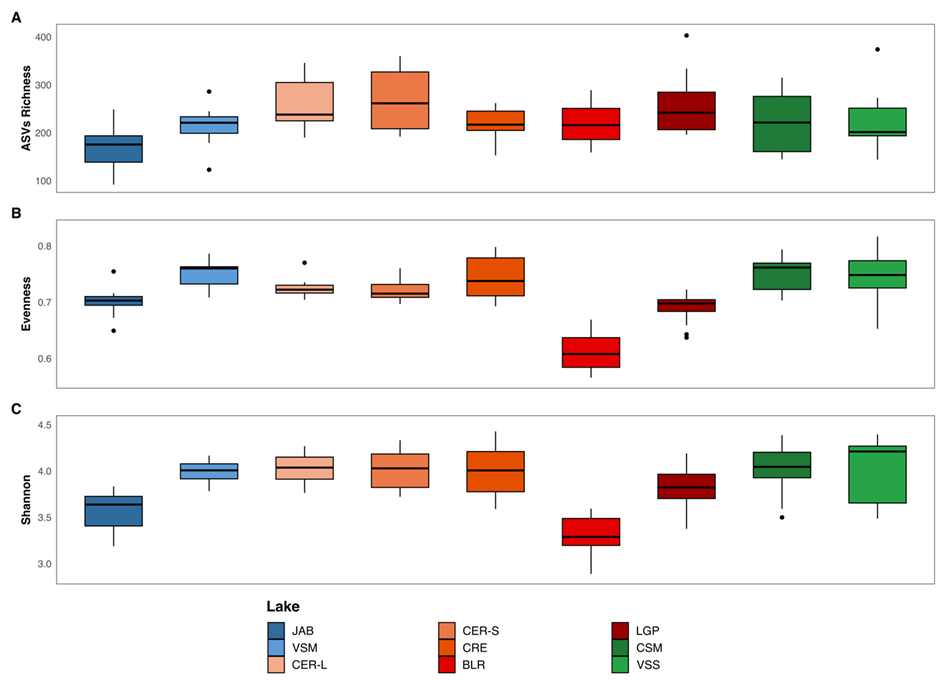
**

**Fig. S5: Prokaryotic taxa-content alpha-diversity indices.** ASV Richness (**A**), Evenness (**B**) and Shannon diversities (**C**) for each lake over the four summer months (104 samples, Table S7). Lakes are colored according to their trophic status (see Fig. 1B).

**
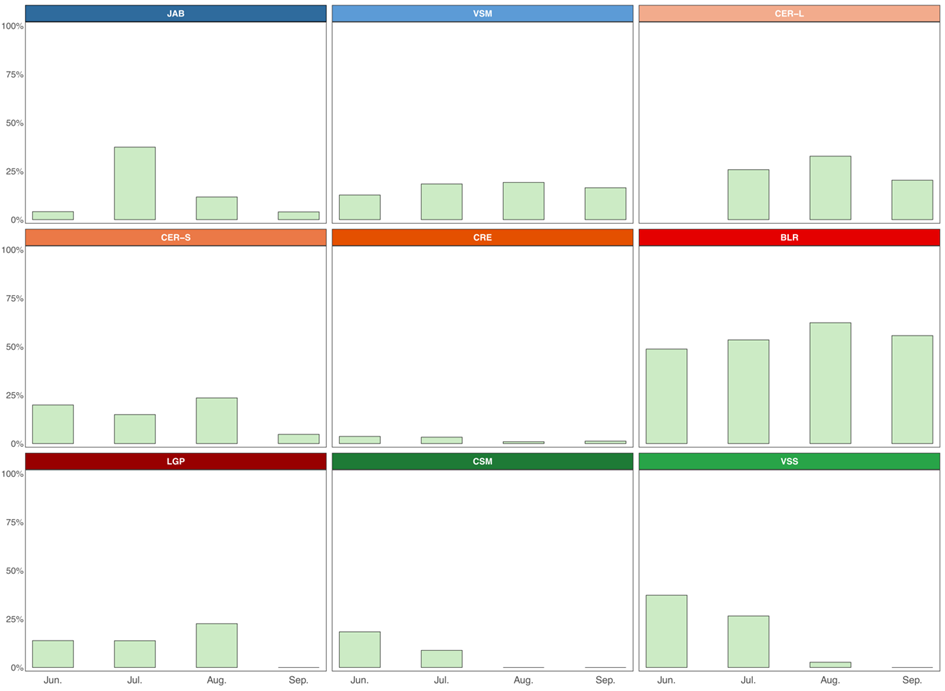
**

**Fig. S6: Relative abundance (%) of *Cyanobium* ASVs over the four summer months.** Median abundance of the *Cyanobium* ASV (Cyanobacteria), as median percentage of total 16S rRNA reads (104 samples). Lakes are colored according to their trophic status (see Fig. 1B).

**
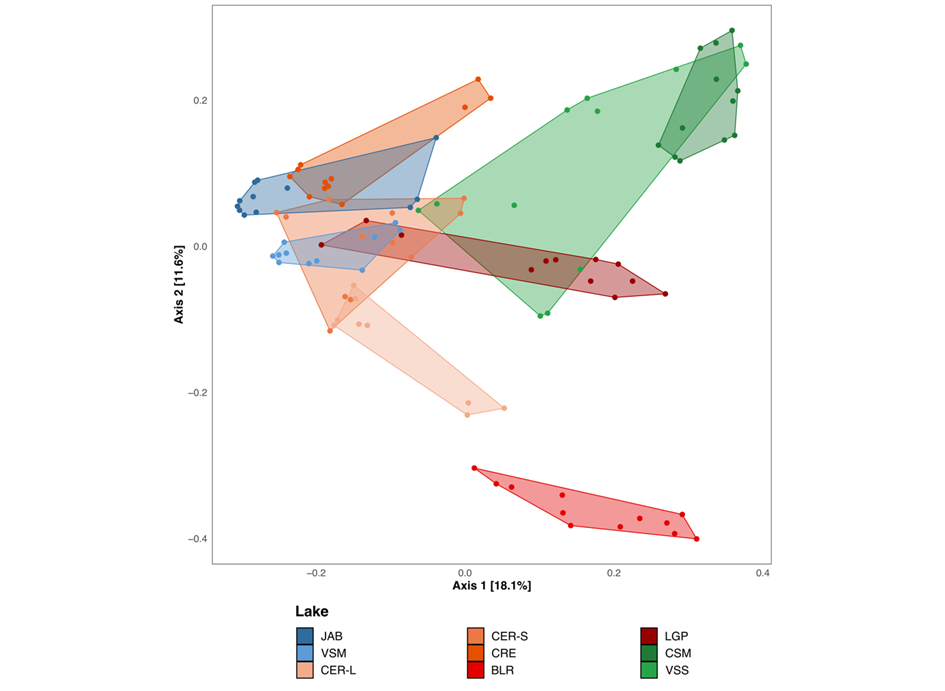
**

**Fig. S7: comparison of lake taxa-contents after exclusion of Cyanobacteria**. PCoA plot (BC dissimilarity), based on ASVs after exclusion of Cyanobacteria ASVs. Polygons represent the maximal area delimited by the samples coordinates of each sample for a lake. Lakes are colored according to their trophic status (see Fig. 1B).

**
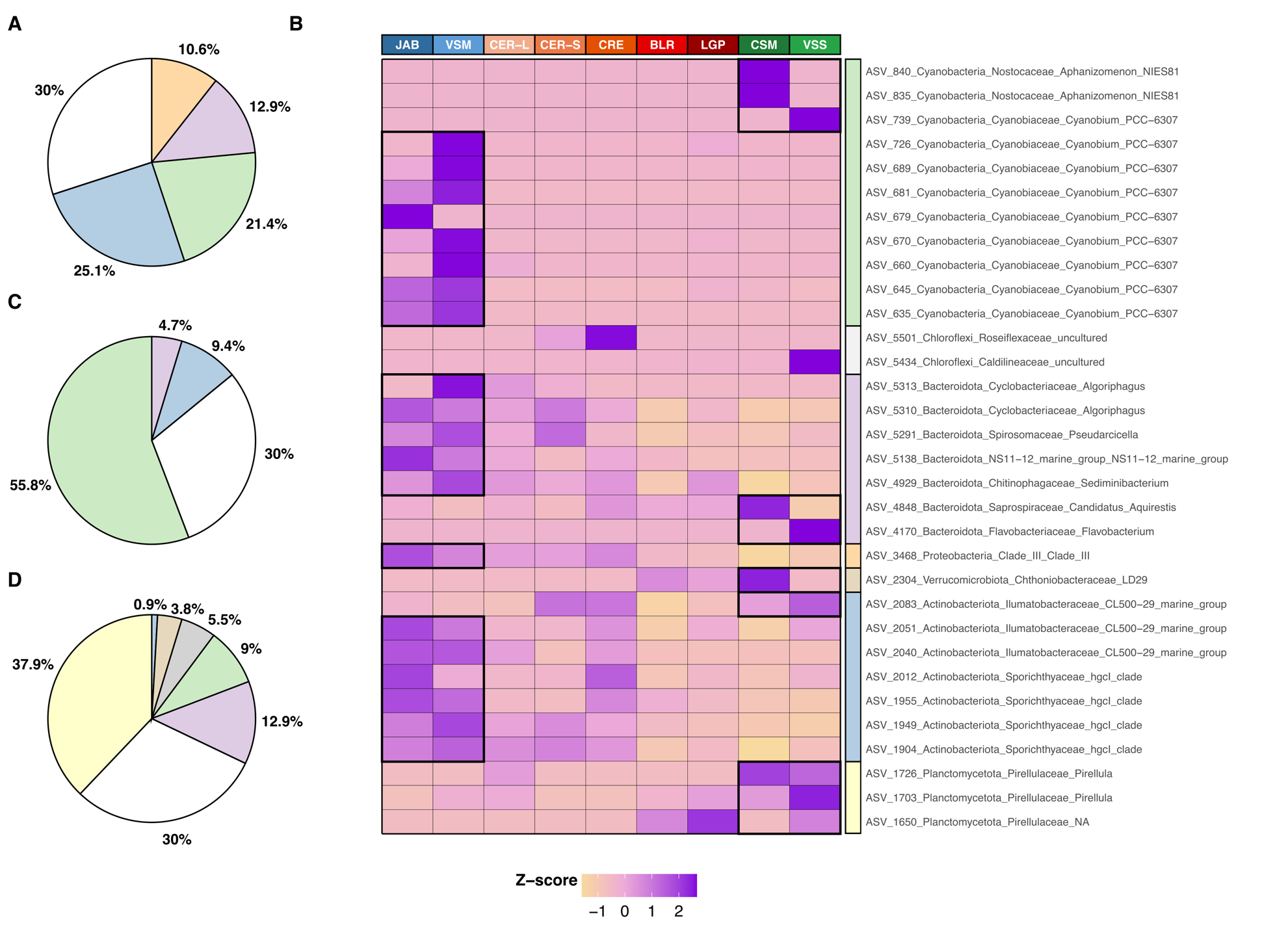
**

**Fig. S8: Set of ASVs that significantly contribute to the ASV-based BC** **dissimilarities between trophic statuses.** Variance (%) explained by the 34 ASVs with a summed abundance over 0.1% (148 ASVs). AVS are represented at the phylum taxonomic rank, **(A)** mesotrophic *vs.* hypereutrophic; **(C)** eutrophic *vs.* mesotrophic; and **(D)** eutrophic *vs.* hypereutrophic comparison. Slices, colored accordingly to the phylum affiliation (see **Fig. 2B)** represent ASVs that accounted for 70% of the cumulated variance while the other 30% are colored in grey (see Material and Methods)**.** **B**: Relative abundance (Z-score) of the 34 ASVs (of the 0.1% of total reads) significantly explaining the BC dissimilarities between at least one pairwise trophic status comparison. Lakes are colored according to their trophic status (see Fig. 1B).

**
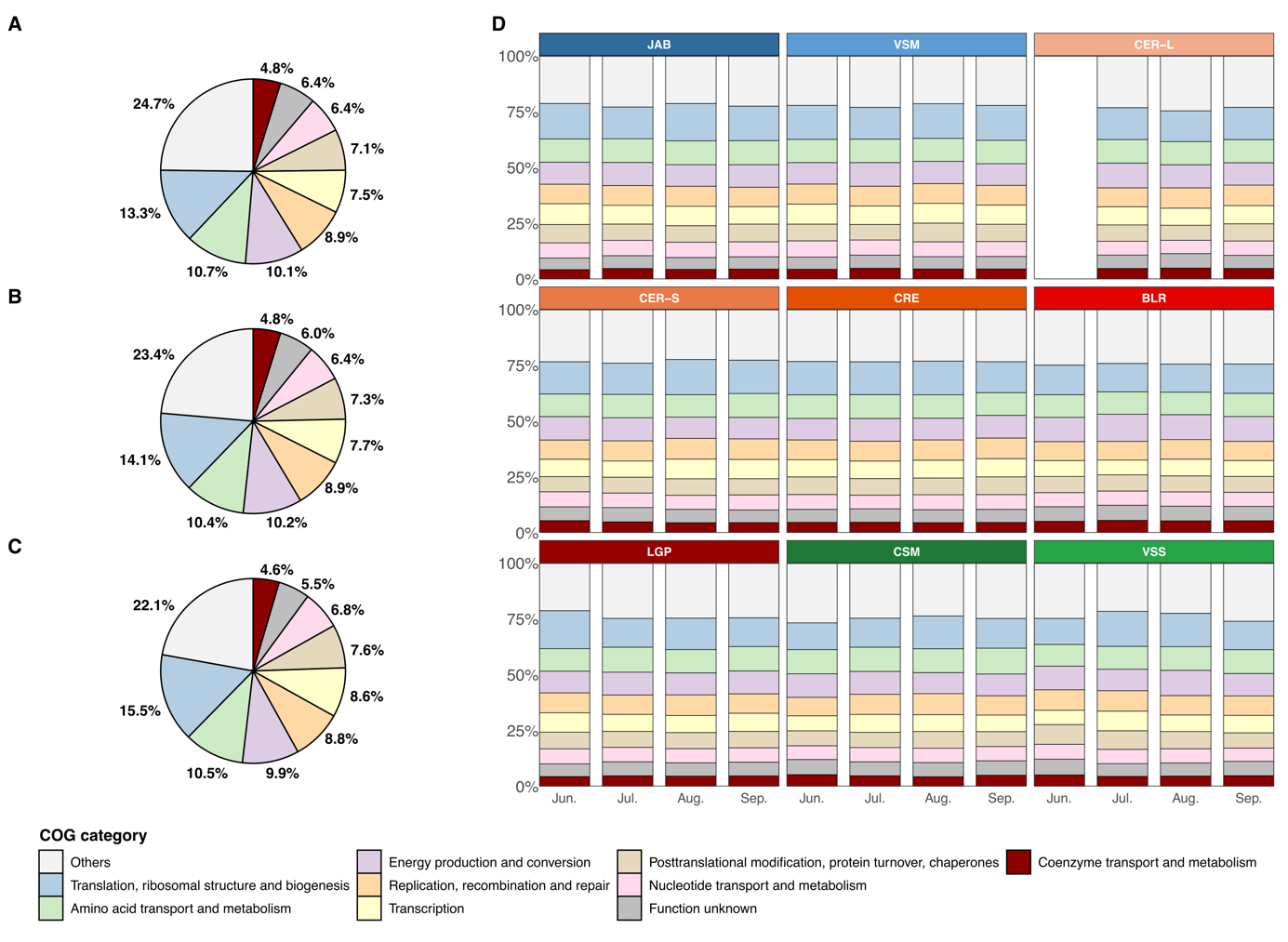
Fig. S9: Prokaryotic gene-content** **composition.** Clusters of Orthologous Genes (COG) categories displayed for each trophic status (**A**: hypereutrophic; **B**: eutrophic; **C**: mesotrophic), and by lake and months (**D**), as median proportion of the total KO abundance (n=1 per lake for each month, 35 samples). Only the ten most abundant COG categories are displayed (out of 25, see Table S9). Lakes are colored according to their trophic status (see Fig. 1B).

**
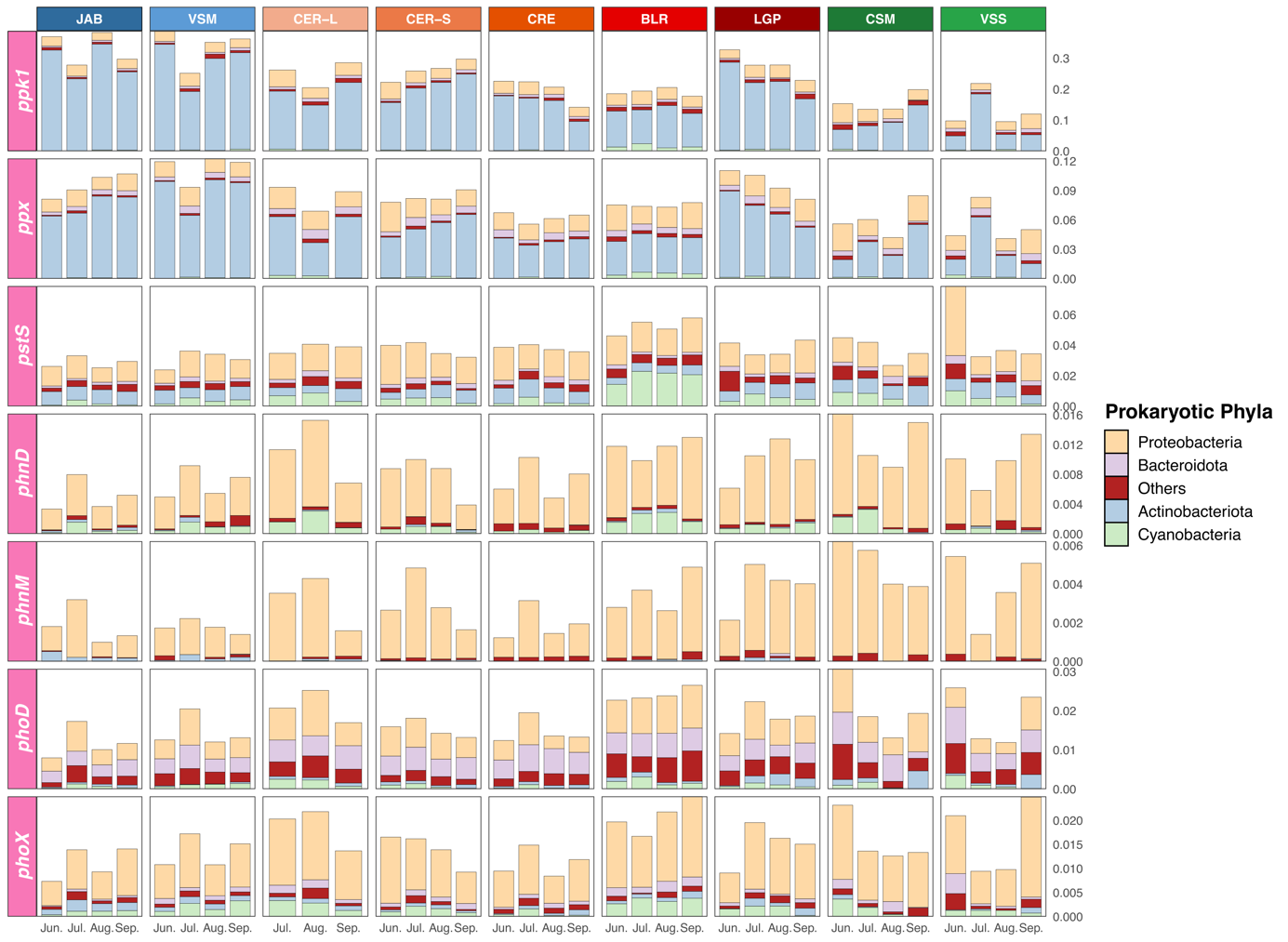
Fig. S10: Abundance and taxonomic affiliation of the BGC marker genes involved in phosphorus metabolism;** over the four months (as percentage of the total KO abundance, from 0 to 100%; Table S3) for each lake (35 samples). Lakes are colored according to their trophic status (see Fig. 1B).

**
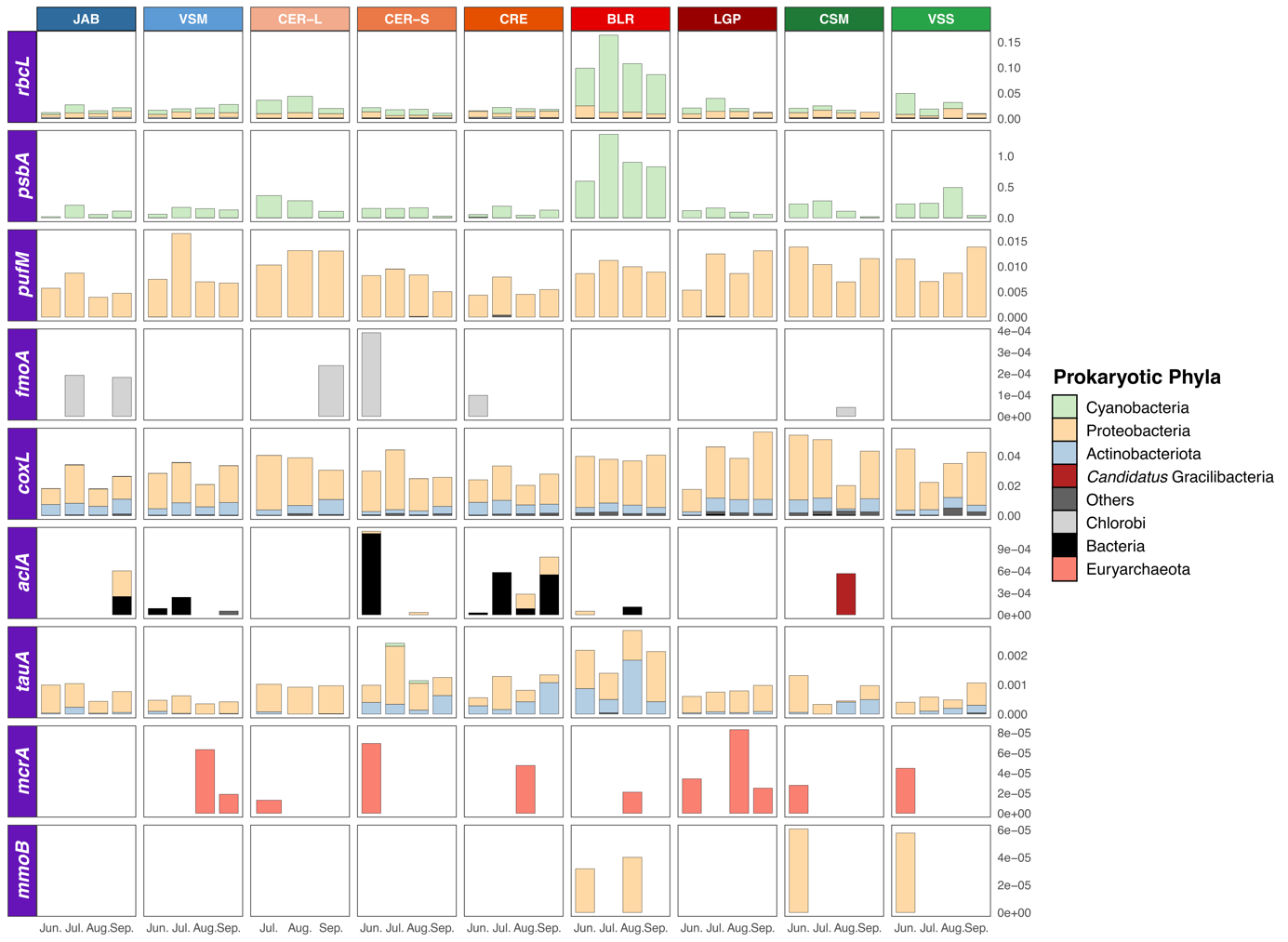
Fig. S11: Abundance and taxonomic affiliation of the BGC marker genes involved in carbon metabolism;** over the four months (as percentage of the total KO abundance, from 0 to 100%; Table S3) for each lake (35 samples). Lakes are colored according to their trophic status (see Fig. 1B).

**
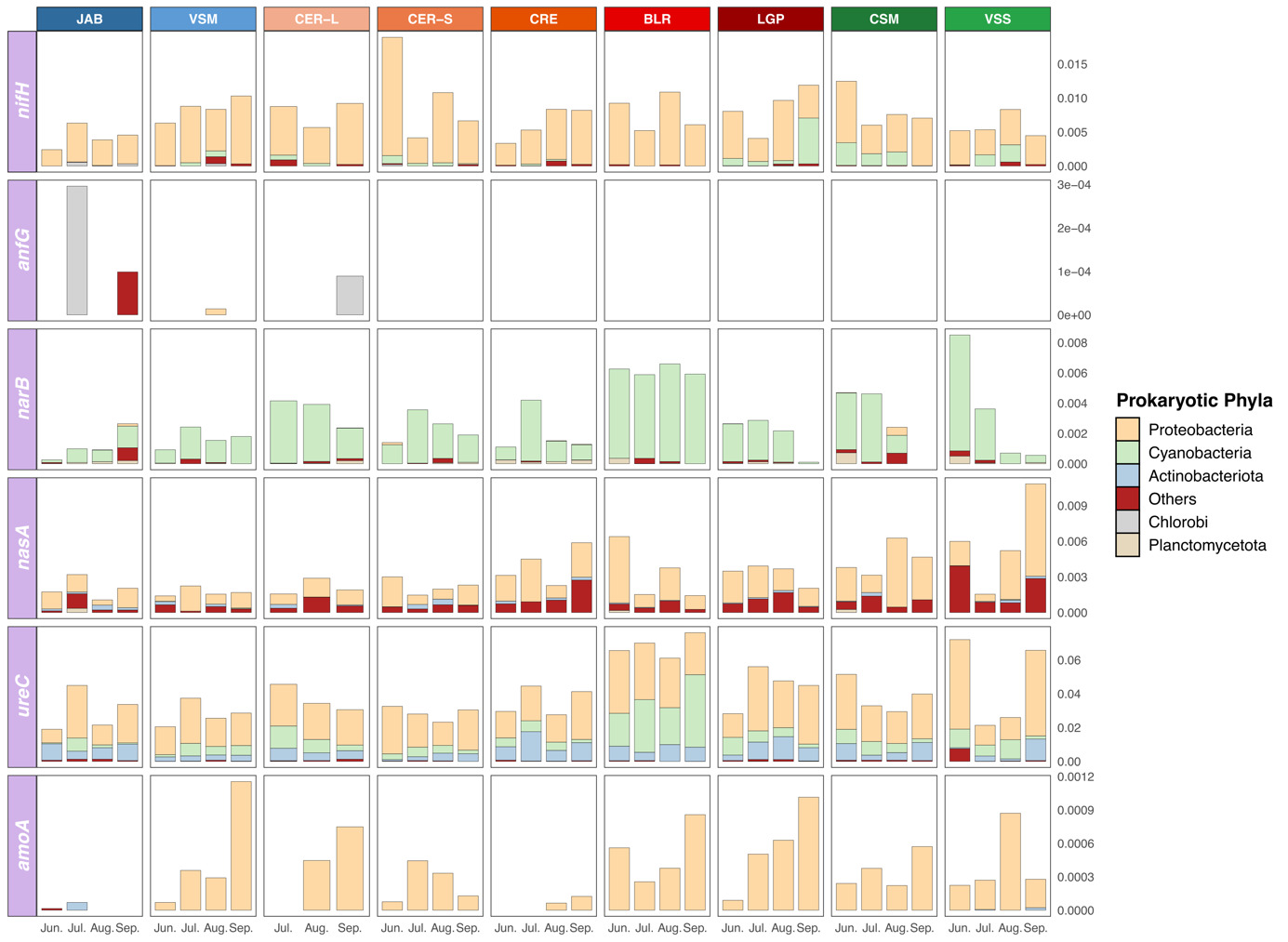
Fig. S12: Abundance and taxonomic affiliation of the BGC marker genes involved in nitrogen metabolism;** over the four months (as percentage of the total KO abundance, from 0 to 100%; Table S3) for each lake (35 samples). Lakes are colored according to their trophic status (see Fig. 1B).
